# Topical Losartan in the Management of Corneal Scarring from Trauma and Infection: A Case Series

**DOI:** 10.1159/cop/acaag023

**Published:** 2026-10-08

**Authors:** Hueyjong Shih, Emma C. Davies

**Affiliations:** a Department of Ophthalmology, Harvard Medical School, Boston, MA, USA; b Cornea and External Disease Service, Massachusetts Eye and Ear, Boston, MA, USA

**Keywords:** Topical losartan, Corneal scarring, Corneal ulcer, Corneal laceration

## Abstract

**Introduction:**

Losartan inhibits ERK-mediated signaling in the noncanonical transforming growth factor β (TGFβ) pathway, which may play a role in corneal fibrosis. Rabbit studies have shown that topical losartan can reduce stromal scarring, fibrosis, and myofibroblast formation after corneal injuries and other clinical insults. Its use in humans has since been reported for corneal scarring and haze following LASIK, corneal cross-linking, and herpes simplex and varicella-zoster virus infections. Here, we present several additional clinical scenarios in which topical losartan has demonstrated potential efficacy in reducing corneal scarring.

**Case Presentations:**

Cases which improved with topical losartan included a full thickness corneal laceration from an open globe injury, four cases of *Pseudomonas* contact-lens related corneal ulcers, one case of a likely herpetic-related corneal ulcer, and one case of a full-thickness *Fusarium* fungal ulcer. Topical losartan was initiated between 2 weeks to over 10 months after the initial corneal insult, with most patients starting treatment within one to 2 months of presentation. No patients experienced any significant side effects from topical losartan use.

**Conclusion:**

Topical losartan may be an effective treatment for corneal scarring secondary to trauma or infection either following or in conjunction with corticosteroid therapy. Patients with such scarring should be considered for inclusion in future clinical trials which investigate the efficacy and safety of topical losartan treatment.

## Introduction

Losartan inhibits the activation of extracellular signal-regulated kinase (ERK) mediated signal transduction in the noncanonical transforming growth factor β (TGFβ) pathway [1]. Animal studies, mainly performed in rabbits, have suggested that topical losartan decreases stromal scarring fibrosis and/or myofibroblast generation after a wide range of clinical insults, including Descemet’s membrane-endothelial excision, alkali burn injury, photorefractive keratectomy, blast-injury simulating irregular phototherapeutic keratectomy, and acute corneal incisions [2–8].

It is hypothesized that losartan treatment of corneal stromal fibrosis occurs in a two-step process [1]. Injuries to the cornea violate the epithelial basement membrane (EBM) and allow TGFβ1 and TGFβ2 from the tears and epithelium to enter the corneal stroma and stimulate the development of fibroblasts and fibrocytes into myofibroblasts [1]. These myofibroblasts are maintained by ongoing TGFβ signaling and produce disordered collagens which contribute to the visible stromal opacity [1]. Topical losartan penetrates the corneal stroma and blocks ERK-1/2 activation in the noncanonical TGFβ pathway, leading to myofibroblast apoptosis, reabsorption and reorganization of disordered collagens by corneal fibroblasts, and regeneration of the EBM which regulates TGFβ1/2 entry into the stroma once again [1].

The first human case of losartan being used to treat corneal scarring occurred in a patient with corneal scarring fibrosis after LASIK surgery complicated by a free cap and severe diffuse lamellar keratitis [9]. Since then, it has been used in humans to treat severe corneal haze after epithelium-off corneal cross-linking and corneal opacities caused by herpes simplex virus (HSV) and varicella-zoster virus (VZV) [10, 11].

This case series highlights several other clinical scenarios in our practice where topical losartan has proven to be efficacious. In each case, patients elected to proceed with off-label use of topical losartan after being informed of the risks, benefits, and alternative treatment options. Patients were also informed that use of topical losartan to treat corneal scarring had previously been described in peer-reviewed case reports and presented at ophthalmic conferences. Most patients had exhausted conventional treatment options for corneal scar formation, including topical prednisolone acetate and observation for potential spontaneous improvement, and were either hesitant to pursue or not candidates for corneal transplantation. The CARE Checklist has been completed by the authors for this case series and is attached as online supplementary material (for all online suppl. material, see https://doi.org/10.1159/cop/acaag023). We present improvement in stromal scarring in one case of a full-thickness corneal laceration from an open globe injury, four cases of *Pseudomonas* contact-lens related corneal ulcers, one case of a likely herpetic-related corneal ulcer, and one case of a full-thickness *Fusarium* fungal ball corneal ulcer.

## Case Presentations

### Case 1

A 40-year-old male sustained a full-thickness corneal laceration (Zone 1 open globe injury) to his left eye due to penetration with a high-speed metallic foreign body while grinding metal. At presentation, the patient’s visual acuity (VA) was count fingers (CFs) at face and exam showed an 8 mm paracentral corneal laceration with lens violation and anterior lens capsule rupture. He required an open globe repair with lensectomy and anterior vitrectomy. At the time of presentation to cornea clinic, it had been 10 months since his open globe repair surgery. He reported “frosty” vision with ghosting of images and persistent monocular diplopia. Corneal topography showed extensive (approximately 4.7 diopters (D)) irregular astigmatism from corneal scarring and slit-lamp exam revealed full-thickness stromal whitening (Fig. 1a). The patient was hesitant to pursue full-thickness corneal transplant and elected to proceed with off-label use of topical losartan six times daily for 3 months to potentially improve his corneal scar.

**Fig. 1.**
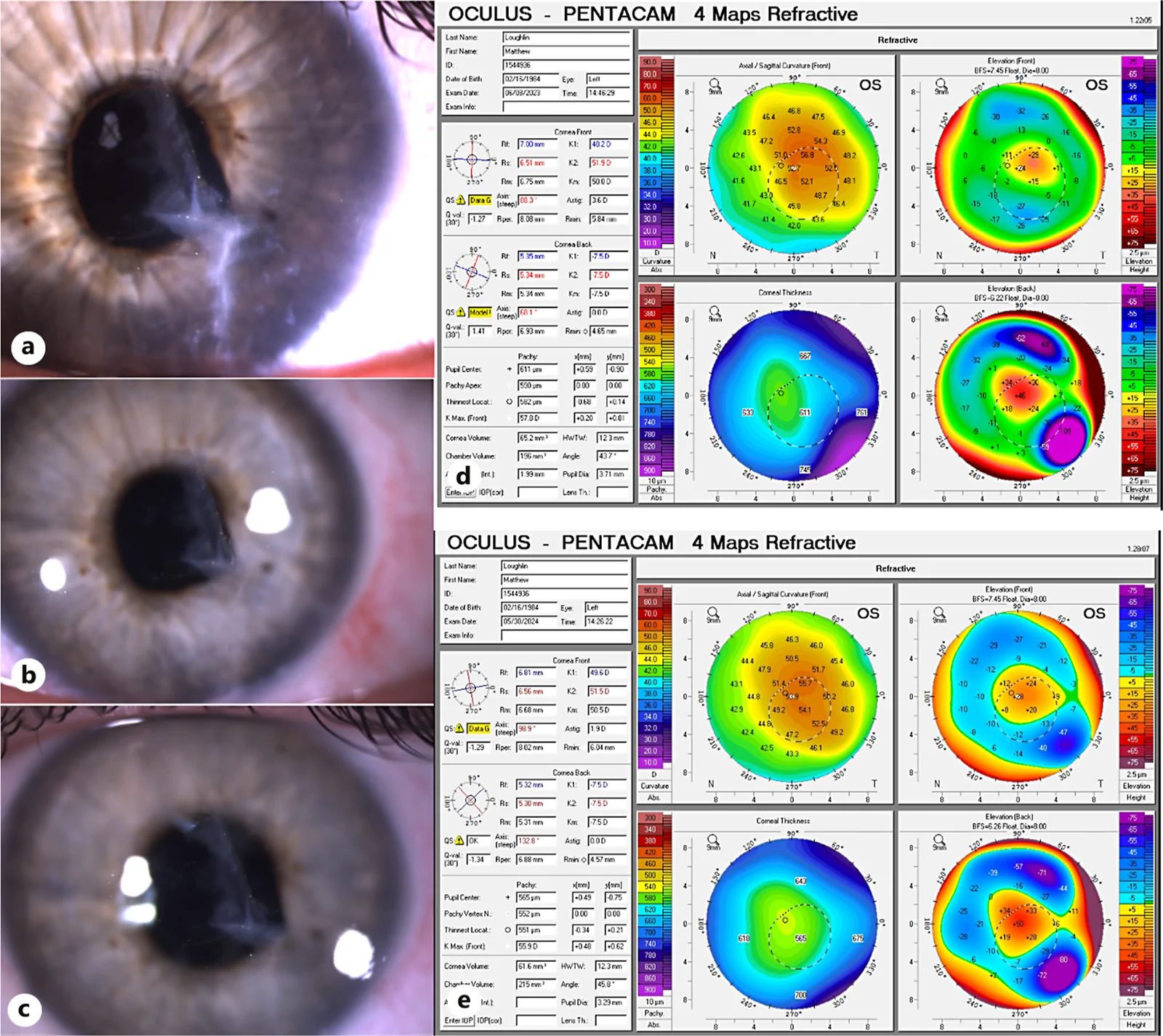
Full-thickness corneal laceration-related corneal scarring. **a** Slit-lamp photograph of the left cornea of case 1 with traumatic full-thickness corneal laceration-related corneal scarring before treatment with topical losartan and 10 months after initial open globe repair surgery. **b** Slit-lamp photograph of the patient’s left eye after losartan six times daily for 3 months and a prednisolone forte taper (four times daily for 1 week, then tapering by one drop each week for 4 week total course). **c** Slit-lamp photograph of the patient’s left eye after losartan six times daily for an additional 3 months. **d** Pentacam corneal topography of the patient’s left eye pretreatment. **e** Pentacam corneal topography of the patient’s left eye after 2 rounds of losartan treatment (3 months each).

The patient returned to clinic 5 months later with significant improvement in corneal scarring. Exam showed faded stromal whitening surrounding his previous irregular full-thickness corneal laceration (Fig. 1b). His VA was 20/25 OS. He was continued on a repeat course of losartan six times daily in the left eye for 3 months to determine if any more improvement could be achieved (Fig. 1c). At the most recent visit, 2 years from the original injury, he requested a third trial of losartan given his level of improvement from previous trials. At this visit, K_max_ on corneal topography decreased from 57.8D to 55.9D (Fig. 1d, e).

### Case 2

A 16-year-old female contact-lens wearer presented with left eye pain and photophobia. At presentation, the patient’s VA was hand motion (HM) in the left eye. Slit-lamp exam showed a central 2.4 mm (vertical (V)) × 1.8 mm (horizontal (H)) soupy corneal ulcer with a 1.6 mm (V) × 1.4 mm (H) central epithelial defect and a 1 mm hypopyon OS. The patient was started on fortified vancomycin every 1 h, fortified tobramycin every 1 h, and cyclopentolate 1% four times per day. After corneal cultures were positive for *Pseudomonas aeruginosa*, the patient was also started on prednisolone acetate 1% twice a day. Over the course of 1 month, antibiotic drops and steroids were tapered and cycloplegia was stopped. The corneal infiltrate and epithelial defect improved and the hypopyon resolved, leaving a central corneal scar in the left eye (Fig. 2a). VA had improved to 20/70 + 2 OS, pinhole 20/30-1 OS, and the corneal exam now showed an irregular trapezoidal shaped anterior and posterior stromal whitening patch in a zone measuring 2.9 mm (V) × 2.5 mm (H) with 15% thinning at site, no infiltrate, and no epithelial defect.

**Fig. 2.**
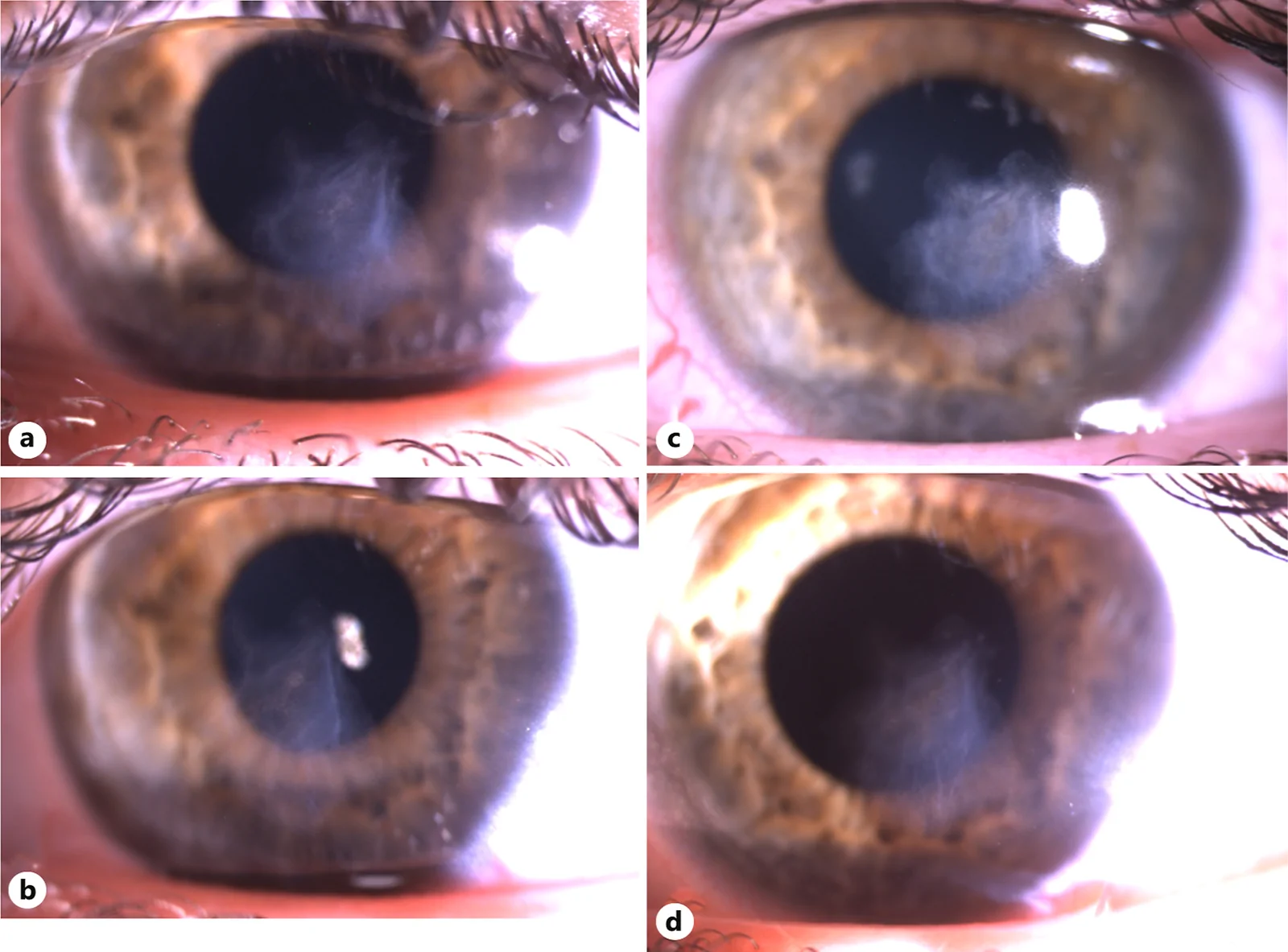
*Pseudomonas aeruginosa* corneal ulcer-related corneal scarring. **a** Slit-lamp photograph of the left cornea of case 2 with *Pseudomonas aeruginosa*–related corneal scarring prior to losartan use. **b** Slit-lamp photograph of the patient’s left eye after 1 month of using losartan 6 times daily with a prednisolone acetate taper. **c** Slit-lamp photograph of the patient’s left eye after not using losartan consistently. The corneal scar was measured to be larger at this visit. **d** Slit-lamp photograph of the patient’s left eye after restarting losartan 6 times daily with a prednisolone forte taper for 6 weeks. The corneal scar was measured to be smaller at this visit and Losartan was stopped.

The patient elected to proceed with off-label use of topical losartan six times daily to improve her corneal scar. One month after starting topical losartan, the patient reported that her vision was improving and she noticed less glare and halos in her vision. VA was 20/40 OS, pinhole 20/30-2 OS. She reported no side effects of the topical losartan eye drops. Exam showed a very faded irregular trapezoidal shaped anterior and posterior stromal whitening patch in a zone measuring 2.3 mm (V) × 2 mm (H) just inferotemporal to central visual axis, 5–10% thinning at site, with no infiltrate, no epithelial defect, and no corneal edema (Fig. 2b). At this visit, topical prednisolone drops were stopped, and the patient was continued on topical losartan six times a day.

At a follow-up visit around 1 month later, the patient reported that she had not used the losartan drops as consistently in the past 2 weeks. Repeat VA was 20/30-2 OS, pinhole 20/20-2 OS. Exam showed a worsened central corneal scar now measuring 4.3 mm (V) × 2.5 mm (H) with iron deposition at the site (Fig. 2c) and a prednisolone taper was restarted in addition to restarting regular use of topical losartan.

Six weeks later, the patient reported overall stable vision. VA was 20/40 OS, pinhole 20/25-2 OS. Exam showed an improved corneal scar now measuring 3.1 mm (V) × 2.1 mm (H) with iron deposition at site (Fig. 2d). Losartan was stopped, as the patient had completed a 3-month course, and pred forte was slowly tapered given prior worsened stromal whitening when the patient came off the steroid previously. Two months later, the patient had no change in vision and her corneal scar was stable off all drops.

### Case 3

A 17-year-old male contact-lens wearer presented with left eye pain, difficulty opening his eyelid, yellow discharge, and sensitivity to light for 2 days. The patient’s VA was 20/200 OS, pinhole to 20/50 OS. On exam, there was a large purulent paracentral superior corneal ulcer measuring 3 mm (V) × 4.5 mm (H) with thinning, 0.7 mm hypopyon, and 3-4+ anterior chamber (AC) cell OS. He was started on topical fortified vancomycin and fortified tobramycin every 1 h and corneal cultures grew *Pseudomonas aeruginosa*. At his initial visit to the cornea clinic 2 days after presentation, VA was CF at 1 feet, pinhole to 20/40-2 OS. The ulcer had decreased in size to 2.5 mm (V) × 2.9 mm (H) and the infiltrate measured 4.2 mm (V) × 4.9 mm (H) (Fig. 3a). The hypopyon was resolved but there remained 3-4+ AC cell OS. He was continued on his fortified antibiotic drops. Four days later, the corneal infiltrate had decreased in size, now 3.5 mm (V) × 3 mm (H) with 2+ AC cell OS. Fortified tobramycin was decreased to every 2 h, fortified vancomycin was stopped, moxifloxacin was added at every 2 h, and prednisolone acetate was added on at twice a day OS (Fig. 3b). One week later, the patient returned to clinic with VA 20/50-1 OS, pinhole to 20/25-2 OS. The corneal infiltrate had turned into a prominent corneal scar with anterior and posterior stromal whitening in zone measuring 4.4 mm (V) × 2.8 mm (H) from 1 o’clock to 3 o’clock paracentrally, with no epithelial defect, and 5% thinning at the site. Moxifloxacin and fortified tobramycin were decreased to six times daily, prednisolone acetate was continued, and topical losartan six times daily was started.

**Fig. 3.**
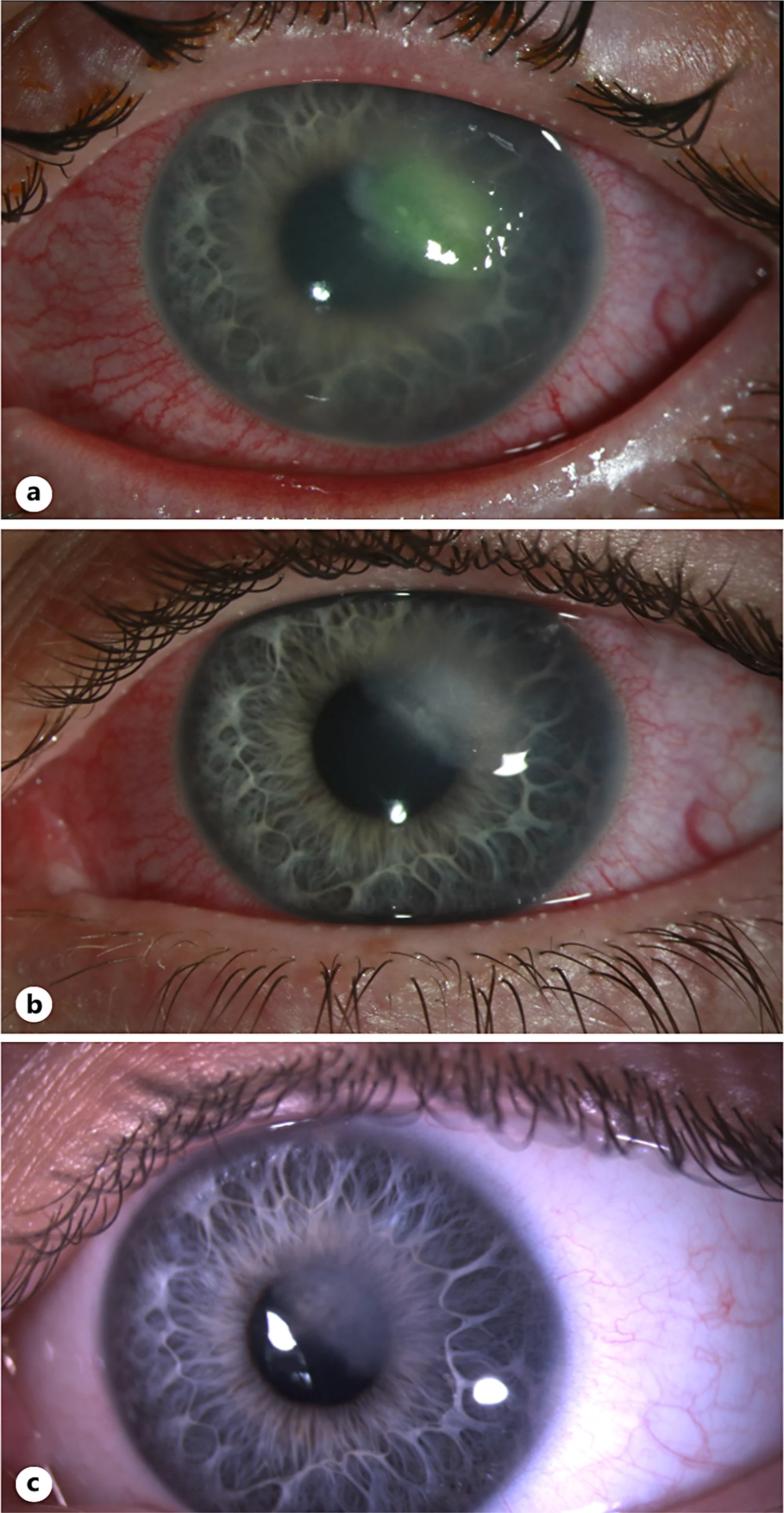
*Pseudomonas aeruginosa* corneal ulcer-related corneal scarring. **a** Slit-lamp photograph of the left cornea of case 3 with *Pseudomonas aeruginosa*–related corneal infiltrate prior to starting topical losartan. The patient was continued on fortified antibiotic drops at this visit. **b** Slit-lamp photograph of the patient’s left eye 4 days later with decreased size of corneal infiltrate. Prednisolone acetate was started two times daily this visit. **c** Slit-lamp photograph of the patient’s left eye 1.5 months after treatment with losartan 6 times daily with concurrent prednisolone acetate therapy.

Two weeks later, the patient’s VA remained at 20/50-1 OS, pinhole to 20/25 + 1 OS. The prominent corneal scar had decreased in size to 4 mm (V) × 2 mm (H) with ∼1% thinning at the site. Moxifloxacin and fortified tobramycin drops were discontinued, prednisolone was increased to three times daily, and losartan six times daily was continued.

The patient returned 1 month later with improved visual acuity to 20/40-3 OS, pinhole to 20/20-2 OS. The corneal scar remained stable (Fig. 3c) and the patient elected to continue with observation rather than continuing prednisolone acetate and losartan treatment.

### Case 4

A 66-year-old male contact-lens wearer presented with foreign body sensation, discomfort, swelling, and clear mucous discharge for 3 days. The patient had been wearing his contact lenses for 2 weeks straight without removing them or cleaning them. At presentation, the patient’s VA was HM OS, with no improvement on pinhole and on exam there was a 4.2 mm × 4.5 mm epithelial defect with infiltrate in the central visual axis (Fig. 4a). He was started on topical fortified vancomycin and fortified tobramycin every 1 h. Corneal cultures grew *Pseudomonas aeruginosa*.

**Fig. 4.**
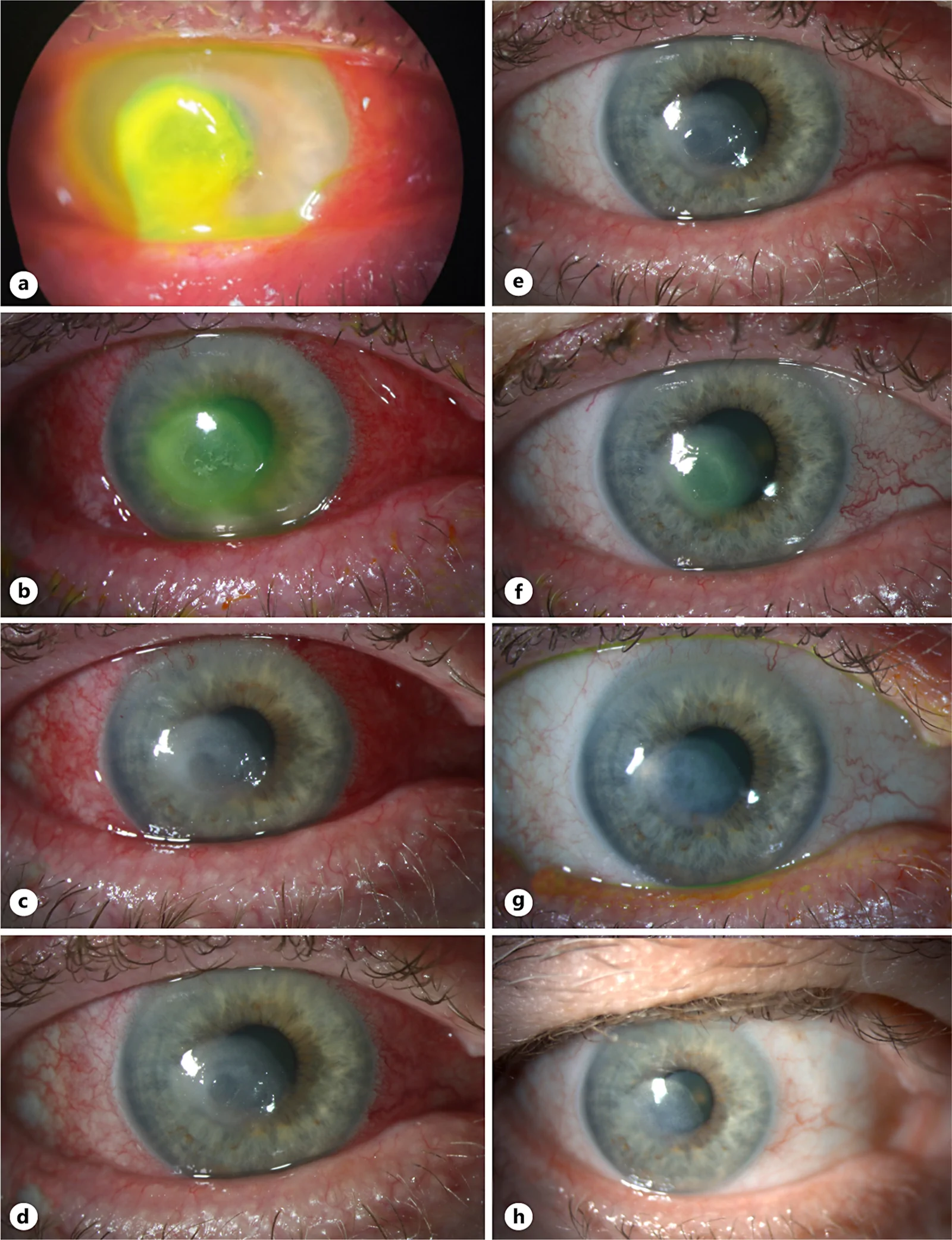
*Pseudomonas aeruginosa* corneal ulcer-related corneal scarring. **a** Slit-lamp photograph of the left cornea of case 4 with *Pseudomonas aeruginosa*–related corneal infiltrate prior to starting fortified antibiotics. **b** Slit-lamp photograph of the patient’s left eye 2 days later showing a new inferior infiltrate. **c–f** Sequential slit-lamp photographs of the patient’s left eye over the next 2 weeks showing a decrease in the size of the epithelial defect and area of corneal infiltrate. Topical losartan was started 8 weeks post-presentation. **g** Slit-lamp photograph of the patient’s left eye 1 month after starting topical losartan 4 times daily (patient was recommended to start topical losartan 6 times daily but did not use it as recommended). The corneal scar was noted to be improving. **h** Slit-lamp photograph of the patient’s left eye 1 month later after adhering to topical losartan at the recommended dosage of 6 times daily.

Two days later, the patient’s VA was 20/125 OS. The ulcer had changed in size to 3.7 mm (V) × 5.3 mm (H) epithelial defect with infiltrate, and there was a new 1.5 mm × 2.1 mm inferior infiltrate as well (Fig. 4b). The patient was started on valtrex and continued fortified antibiotic drops.

Over the next 2 weeks, the patient’s fortified vancomycin and fortified tobramycin drops were tapered and then stopped. Moxifloxacin four times daily, prednisolone acetate two times daily, ciloxan ointment nightly, and doxycycline 100 mg orally daily were added to his medication regimen. A punctal plug was placed for optimization of ocular surface lubrication. The epithelial defect and area of infiltrate continued to decrease with each visit (Fig. 4c–f).

He continued this regimen until 8 weeks post-presentation but was hoping more could be done to reduce the scarring and “fogginess” in vision OS and expressed interest in trying off-label use of losartan. At this visit, his VA had improved to 20/30-1 OS, pinhole to 20/25-2 OS. His exam was now notable for faded anterior and posterior stromal whitening in a zone measuring 3.2 mm (V) × 2.1 mm (H) centrally with 5% thinning, and an epithelial defect measuring 0.9 mm (V) × 0.6 mm (H) at the center of the site. He was instructed to start on losartan six times daily, but the patient used losartan only four times daily in addition to his prior regimen.

One month later, his VA remained stable at 20/30 OS, pinhole to 20/25 OS. His exam showed an improving corneal scar with no epithelial defect and the patient reported slight improvement in his vision with less “fogginess” (Fig. 4g). Moxifloxacin and ciloxacin ointment were discontinued, and topical losartan was increased to six times daily.

At his latest visit 1 month later, his VA improved to 20/25-2 OS, pinhole to 20/25 OS and his corneal scarring continued to improve (Fig. 4h). The patient reported stable visual symptoms and at this visit, prednisolone was tapered and then stopped and losartan was discontinued.

### Case 5

A 31-year-old male contact-lens wearer presented with redness, blurry vision, and white discharge OS. At presentation, the patient’s VA was 20/70-2, pinhole to 20/60 + 2 OS. On exam, there was a 4 mm diameter ulcer with similar size epithelial defect involving the central visual axis. There was 1+ AC cell and no hypopyon. He was started on topical fortified vancomycin and fortified tobramycin every 1 h, oral vitamin C, and oral doxycycline. Corneal cultures grew *Pseudomonas aeruginosa*.

Two days later, the patient’s VA was CF at two feet, and the ulcer was similar in size with 20–30% central thinning noted. Ciloxan ointment was started, every 2 h fortified tobramycin was tapered, and fortified vancomycin was stopped. Topical prednisolone acetate 1% was started at three times per day. A slit-lamp photo (Fig. 5a) and anterior-segment optical coherence tomography (Fig. 5b) was obtained 6 days after presentation. Over the next 2 weeks, the size of the epithelial defect and infiltrate slowly decreased with each visit.

**Fig. 5.**
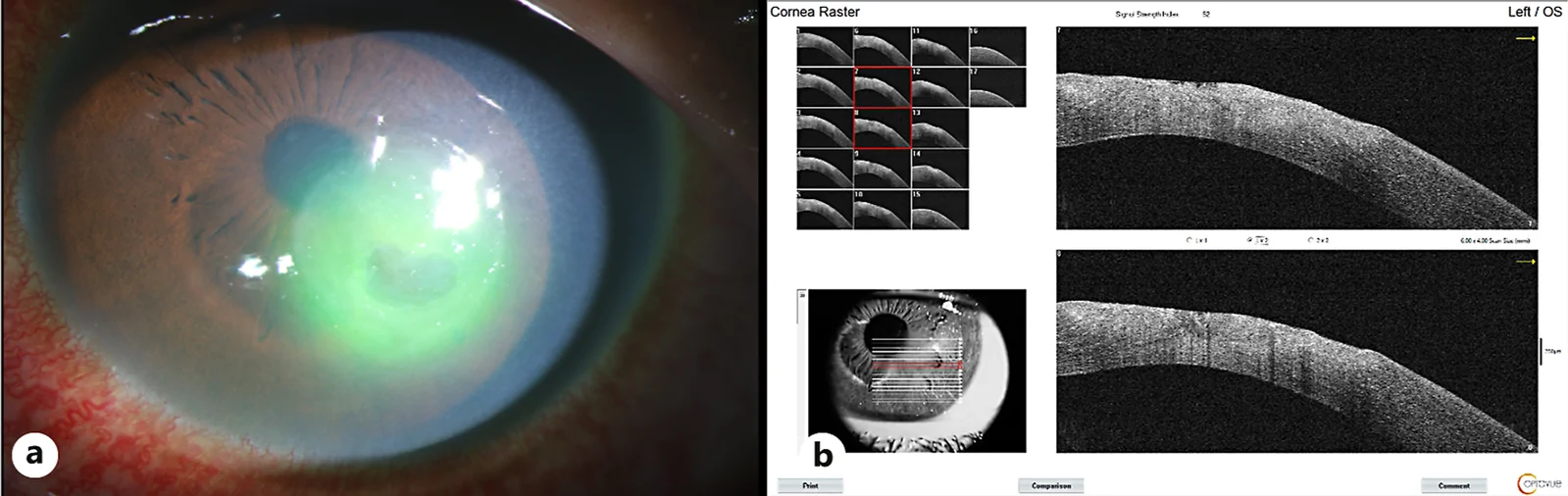
*Pseudomonas aeruginosa* corneal ulcer-related corneal scarring. **a** Slit-lamp photograph of the left cornea of case 5 with *Pseudomonas aeruginosa*–related corneal infiltrate 6 days after initial presentation. **b** Anterior-segment ocular coherence tomography of the left eye showing corneal thinning and stromal involvement of infection 6 days after initial presentation.

At around 1 month after presentation, the patient’s VA had improved to 20/30-2, pinhole to 20/25 OS. At this time, there was anterior and posterior stromal whitening at the previous ulcer site measuring 3.2 mm (V) and 3.8 mm (H) with only 5% thinning. Fortified tobramycin was gradually tapered off, and both Vitamin C and ciloxan ointment were stopped. The patient elected to start losartan 1 drop six times daily in the left eye to try to minimize scarring.

Two weeks later, VA remained stable, and the area of anterior and posterior stromal whitening measured 2.5 mm (V) and 3 mm (H) with similar thinning. At this visit, topical prednisolone was tapered off, and topical losartan was continued for another 6 weeks.

Eight weeks later, the patient noticed subjective improvement in his vision and was now off all drops. The anterior and posterior stromal whitening now measured 2.3 mm (V) and 2.3 mm (H) with stable thinning.

### Case 6

A 46-year-old female contact-lens wearer presented with swelling, pain, redness, tearing, and blurred vision OD. She last wore her contact lenses 2 days prior to presentation with wear time frequently between 10 and 14 h per day. She also had a history of herpes simplex virus oral ulcers, but she had not been taking valtrex prophylaxis. At presentation, the patient’s VA was 20/70-2, pinhole to 20/50-2. On exam, there was a 1.4 mm (V) × 1 mm (H) corneal ulcer inferior to the visual axis with surrounding stromal haze and corneal edema but no KP. There was also 3+ cell, 1+ flare, and fibrin strands in the AC. She was started on topical fortified vancomycin and fortified tobramycin every 1 h as well as valtrex 1g three times a day given her history and concern for herpetic etiology of the corneal ulcer.

At her 1-day follow-up visit, the patient reported that she had a new cold sore on her lip. VA OD now was CF at 2 feet and the corneal ulcer had increased in size to 2.0 mm (V) × 1.3 mm (H) with surrounding stromal haze. The patient was continued on both topical fortified antibiotics every hour for one more day OD then tapered to every 2 h and valtrex decreased to 500 mg orally twice daily.

One week later, VA improved to 20/25-1 OD and the size of the anterior and posterior stromal whitening OD decreased to 1.4 mm (V) × 0.9 mm (H). The decision was made to start prednisolone acetate twice daily OD and decrease fortified antibiotics to four times daily given good control of infection.

Two weeks after presentation, corneal stromal whitening continued to improve OD. Fortified antibiotics were stopped OD and prednisolone acetate was gradually tapered to once per day.

Around 1 month after presentation, corneal stromal whitening and VA remained stable OD; however, patient was still bothered by glare and halos from the remaining stromal whitening (Fig. 6a). The patient elected to start topical losartan six times daily OD and continue prednisolone acetate once daily OD. Two weeks later, the patient reported that she self-discontinued prednisolone acetate due to patient’s concerns that it was making her gain weight and had only been able to use the topical losartan six times daily for 5 days. At this visit, oral valtrex was discontinued due to her completing the course, and she was restarted on prednisolone acetate twice daily OD and losartan six times daily OD.

**Fig. 6.**
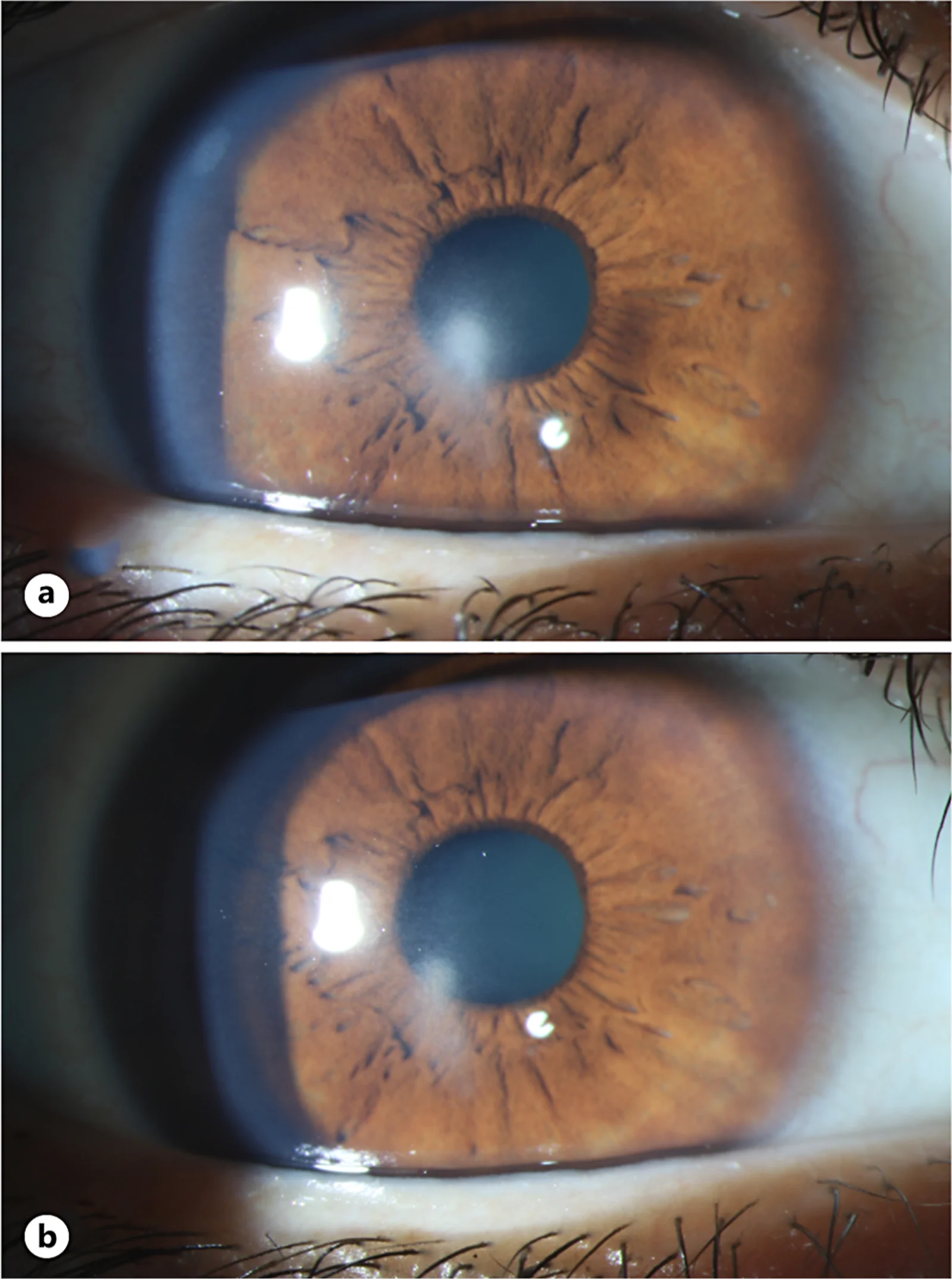
Likely herpetic corneal ulcer-related corneal scarring (**a**) Slit-lamp photograph of the right cornea of case 6 with likely herpetic corneal ulcer-related corneal scarring 1 month after presentation (**b**) Slit-lamp photograph of the patient’s right eye 7 weeks later after using losartan 6 times daily for 5 weeks and inconsistent use of prednisolone acetate.

Five weeks later, the patient returned with a faded corneal scar OD (Fig. 6b) and VA was correctable to 20/20 OD.

### Case 7

A 59-year-old female contact-lens wearer presented to our cornea service for a second opinion of a prior corneal ulcer OS. She reported that she had a contact-lens related ulcer that started 6 weeks ago with some inflammation and was prescribed ciprofloxacin and prednisolone acetate with some improvement. On presentation, VA was 20/100, pinhole to 20/80 OS. There were three punctate areas of epithelial cell loss and a paracentral, “fluffyˮ corneal endothelial infiltrate (Fig. 7a) and 3+ AC cell OS. The patient was cultured and started on fortified vancomycin and fortified tobramycin hourly, cosopt twice a day, valtrex 1 gram twice a day, and erythromycin ointment was increased to three times a day. Prednisolone acetate was stopped.

**Fig. 7.**
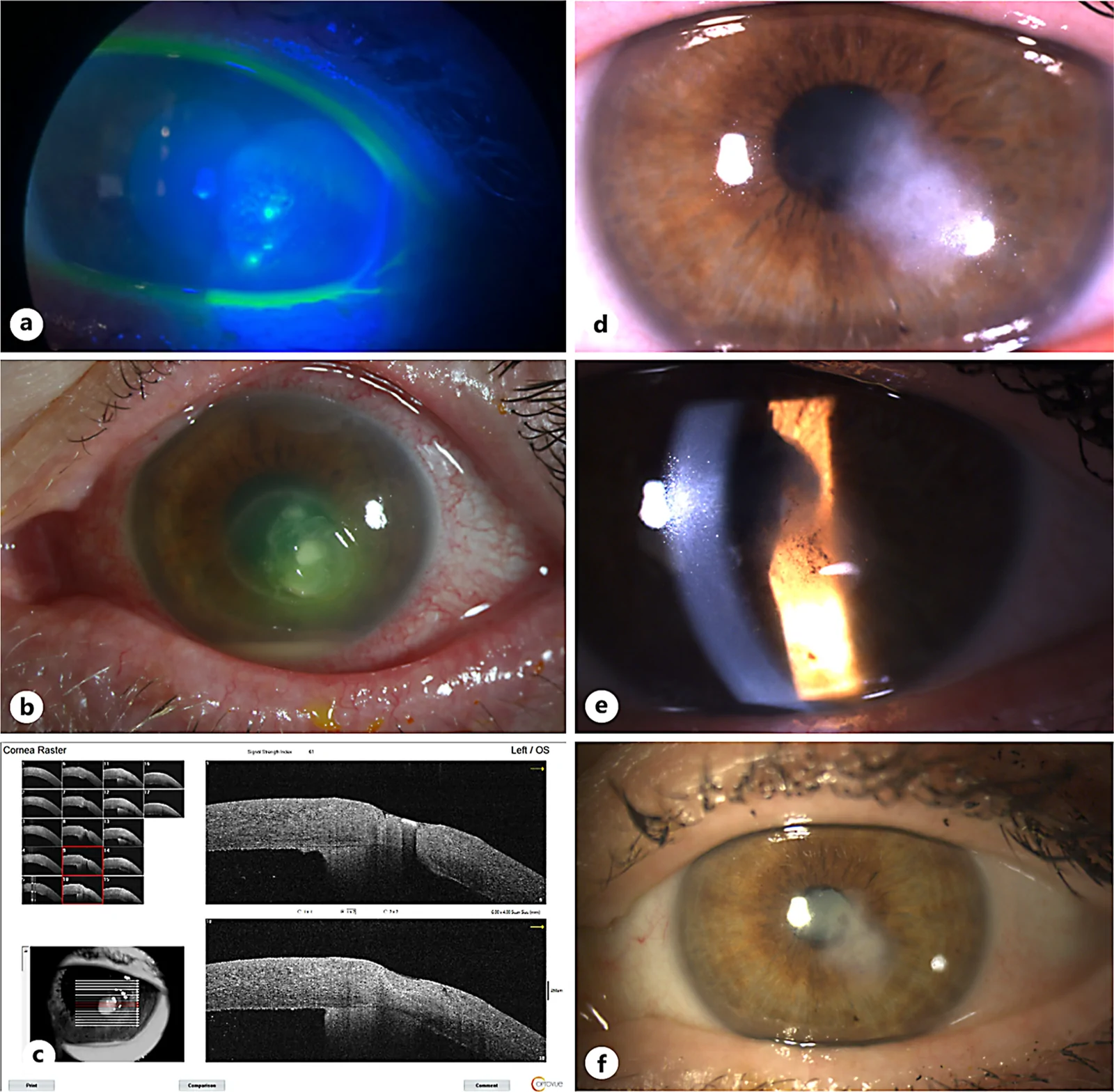
Fusarium corneal ulcer and fungal ball-related corneal scarring (**a**) Slit-lamp photograph of the left cornea of case 7 with Fusarium corneal infiltrate at presentation (**b**) Slit-lamp photograph of the patient’s left eye two later after starting fortified antibiotics, oral valtrex, and erythromycin ointment (**c**) Anterior-segment ocular coherence tomography showing stable corneal thinning 10 days after presentation (**d**) Slit-lamp photograph of the patient’s left eye 3 months after presentation with anterior and posterior stromal whitening and a posterior plaque along the posterior stromal and endothelial surfaces. Topical losartan 6 times daily and FML twice a day was started at this visit. **e** Slit-lamp photograph of the patient’s left eye 3 weeks later showing faded anterior and posterior stromal whitening. FML and topical losartan were continued at the previous dose. **f** Slit-lamp photograph of the patient’s left eye 1 week later showing no anterior whitening and faded posterior stromal whitening.

At a follow-up visit 2 days later, corneal cultures grew rare *Fusarium*. VA was 20/300 and exam revealed a 3 mm (V) × 2.4 mm (H) epithelial defect with disciform-like infiltrate and a 0.6 mm hypopyon (Fig. 7b). Fortified vancomycin and tobramycin were stopped. Natamycin every 1 h was started as well as moxifloxacin four times a day OS.

Over multiple follow-up appointments in the next week, the epithelial defect size continued to decrease to a minimum of 0.5 mm (V) × 0.4 mm (H). Natamycin was tapered down to every 2 h while awake. Anterior-segment optical coherence tomography showed an area of stable thinning (Fig. 7c).

About 1.5 weeks after presentation, VA was 20/60-2 OS, pinhole to 20/40 + 2. However, there was now an endoplaque with overlying central ulceration with 50% thinning, 1-2+ Descemet’s membrane folds, 1 mm hypopyon, 1+ AC cell, and a large epithelial defect OS. Voriconazole 200 mg orally twice daily was started. Moxifloxacin four times daily and polysporin nightly were added prophylactically given large epithelial defect.

One week later, VA declined further to 20/400, pinhole to 20/300 and she had developed a full-thickness fungal ball within the cornea in a zone measuring 3.5 mm (V) × 2.5 mm (H). The epithelial defect had resolved and there were 1+ Descemet’s membrane folds extending from the site. Natamycin was discontinued in favor of amphotericin B every 2 h, and moxifloxacin, and polysporin ointment were stopped given resolution of the epithelial defect.

Over the next month, the fungal ball began to consolidate, leaving anterior stromal whitening and a posterior plaque along the posterior stromal and endothelial surfaces in a zone measuring 3 mm (V) × 1.4 mm (H) paracentrally with endopigment. Over the next few weeks, the area of anterior and posterior stromal whitening remained stable in size. Amphotericin B was decreased to four times daily. Topical steroids to minimize corneal scarring were deferred given concern to not propagate fungus.

At the follow-up visit 3 months after initial presentation, corneal findings remained stable (Fig. 7d). The patient elected to use losartan six times per day to try to reduce corneal scarring. Fluorometholone 0.1% (FML) twice a day was also added given that there was no apparent fungus despite lower antifungal dosing at this visit.

At the next follow-up visit 3 weeks later, there was faded anterior and posterior stromal whitening (Fig. 7e). Amphotericin B was reduced once again to three times daily, and the patient continued topical losartan and FML at the previous dosages.

At the most recent follow-up visit, 1 month later, VA was 20/25-2, pinhole to 20/20. Now, there was no anterior stromal whitening and posterior stromal whitening had faded to an area of 2.4 mm (V) × 1.5 mm (H) (see Fig. 7f). The patient reported subjectively decreased glare and halos from lights in her vision. Amphotericin B was decreased to twice daily and losartan and FML continued.

## Discussion

The seven cases reported here highlight the potential efficacy of topical losartan treatment in reducing corneal scarring from various etiologies such as full-thickness penetrating trauma, bacterial contact lens-related ulcer, likely herpetic corneal ulcer, and fungal contact-lens-related ulcer. In the patients presented, topical losartan was initiated between 2 weeks to over 10 months after the initial corneal insult, with most patients starting treatment within one to 2 months of presentation. The success of losartan in a variety of cases and after various periods of time from corneal injury supports the current hypothesis that fibrotic corneal tissue is dynamic and continuously dependent on TGFβ signaling from the tears and epithelium. This improvement with losartan despite a lengthy period after initial insult has also been described after cases of VZV keratitis, where clearing of the opacity was seen after 3 years [11].

Our patients used topical losartan for different lengths of treatment, ranging from 5 weeks to just over 9 months. The 4 patients who had *Pseudomonas* contact-lens related ulcers used losartan for just between one to 2 months prior to noticing subjective improvement and clinicians noticing objective improvement. Other groups have reported patients requiring topical losartan for more than six to 9 months prior to noticing any improvement in some cases [11]. It is hypothesized that the difference in treatment time is not limited by the apoptosis of myofibroblasts (which occurs within weeks of starting treatment) but the regeneration of defective EBM and removal of disordered extracellular matrix [1, 11]. The comparatively rapid rate at which corneal bacterial ulcers responded to losartan posits the question of whether the EBM either is not as damaged or is able to be repaired more quickly than other etiologies of corneal damage.

One research group which infected rabbit corneas with *Pseudomonas aeruginosa* after epithelial scrape injury and treated the resultant severe keratitis with topical tobramycin noticed that at 1 month, corneas had no detectible EBM lamina lucida or lamina densa, and the central stroma was packed with myofibroblasts [12]. At two to 4 months after keratitis, the EBM had fully regenerated and myofibroblasts disappeared from the anterior 60–90% of the stroma of most of the rabbit corneas [12]. Interestingly, in most of the corneas in that study, the central corneal endothelium and Descemet’s membrane were also damaged by the severe keratitis and in those areas persisted in approximately the posterior 10% of the central stroma [12]. The authors hypothesized that Descemet’s basement membrane modulates TGFβ effects on keratocytes in the posterior cornea similar to the function of the EBM in modulating TGFβ on keratocytes in the anterior stroma [12]. Given that rabbit endothelial cells can proliferate, the authors hypothesized that further follow-up longer than 4 months might have shown less corneal opacities due to regeneration of Descemet’s membrane and apoptosis of posterior myofibroblasts [12]. This study suggests that EBM regeneration and gradual decrease of opacities caused by myofibroblasts should occur regardless of topical losartan usage, and our case report generates the question of whether losartan is able to speed up or facilitate this process given the positive results in a short period of time.

Another question that our case report raises is whether corneal scarring and opacities from different etiologies persist for different amounts of time and why. A recent study investigating persistent corneal opacity and fibrosis following full-thickness corneal injury in rabbits found that between three to 4 months, the EBM was fully regenerated while Descemet’s membrane exhibited incomplete regeneration [13]. This study used Col III expression (a marker of stromal fibrosis after corneal injury) and found that even at three and 4 months after injury, Col III persisted in 30–40% of the posterior stroma [13]. The patient in our case report who sustained a full-thickness corneal injury had a full-thickness stromal scar at 10 months post-injury that was significantly more opacified than the scars of corneal bacterial ulcer patients at one to 2 months post-infection. One hypothesis for this observation, considering the aforementioned studies, is that traumatic injury to Descemet’s membrane further weakens its regenerative capability compared to injury from bacterial infiltration alone. Improvement in these cases seen due to losartan usage could thus be due to a decrease of TGFβ effects in the posterior cornea which leads to posterior myofibroblast fibrosis apoptosis and allows for endothelial-mediated repair of Descemet’s membrane. This hypothesis is supported by the fact that losartan has been shown to penetrate intact corneal epithelium and can reach the entire depth of stroma [2]. The *Fusarium*-related fungal contact-lens related ulcer case presented here had significant endothelial dysfunction during the acute process with improvement of the posterior plaque scarring with losartan use. Endothelial plaques are a typical feature of fungal keratitis and have been hypothesized to represent a sign of hyphae infiltrating Descemet’s membrane [14]. Losartan was started in this case around 3 months after initial presentation, when the posterior plaque along the posterior stroma and endothelial surface remained stable. Fading of the posterior stromal whitening and endoplaque after 3 weeks could indicate normal regeneration of Descemet’s membrane and apoptosis of posterior myofibroblasts at around 3–4 months after injury, which could be expedited by losartan’s effects on decreasing TGFβ signaling in the posterior cornea [12]. Further studies should be undertaken to investigate the involvement and regenerative qualities of the EBM and Descemet’s membrane in corneal insults of varying etiologies, including fungal infections.

All patients in our study used the same frequency and dosage of losartan (0.8 mg/mL six times a day) and did not have epithelial defects when topical losartan was started. It has previously been suggested that clinicians who wish to initiate prophylactic topical losartan to prevent scarring fibrosis in patients with epithelial defects limit the concentration to 0.2 mg/mL six times a day, based on efficacy data from a rabbit alkali burn model [3].

There are several published studies that have shown no significant improvement in corneal opacities with topical losartan use. One study which investigated topical losartan use in the immediate period following a deep corneal radial incision in rabbit eyes showed that topical losartan did not affect the opacity that developed at the incision at 1 month after injury but did decrease the average density of stromal myofibroblasts surrounding the incision [6]. The authors hypothesized that the opacity in the acute phase of an incision is caused by corneal fibroblasts, which are more opaque than keratocytes, and that the opacity should naturally disappear over the next couple months since fibroblasts are transient and naturally undergo apoptosis [6]. Thus, topical losartan would appear to have no effect in the immediate post-injury phase. The opacity should only persist or increase if myofibroblasts are generated in large numbers and extend beyond the incision, which is possibly what was seen in the full-thickness corneal laceration case that we presented. In these cases, the opacity surrounding the corneal lacerations should diminish over time after injury if topical losartan is able to diminish myofibroblast generation and to trigger apoptosis in already generated myofibroblasts.

Another recently published study investigated eight eyes with scarring due to haze after photorefractive keratectomy, collagen cross-linking, viral keratitis, and chronic edema and found no statistically significant differences noted in visual acuity and densitometry values (a quantitative assessment of corneal haze), despite a tendency towards improvement of corneal transparency [15]. One explanation for the disparate findings seen in this study include the fact that topical losartan was only used at a dose of four times a day instead of six, given that the need for refrigeration made compliance difficult and the fact that patients were on other drops. In addition, the indications in this study were mainly epithelial-stromal injuries, while the corneal insults in our study have been shown to involve Descemet’s membrane in animal membranes. Further investigations should be carried out to investigate whether losartan’s effects are more pronounced in etiologies of corneal scarring where Descemet’s membrane is involved.

Our case study has several limitations. We present only a small number of cases with no controls where corneal scarring was trended without losartan use. Admittedly, such a control would be quite challenging to obtain as it would require the rare patient with bilateral corneal insults of the same mechanism, similar size, and similar corneal depth involved. Losartan drop usage was also variable in our patients, with 2 patients using it less than the recommended 6 times daily dose for a portion of the treatment duration. In addition, steroid drops were often concurrently used so it is difficult to identify the effects of steroids compared to losartan itself on the corneal fibrosis. An ideal randomized trial would involve using no steroids, only steroids, steroids and losartan, and losartan only on a wide range of corneal insults. Reassuringly, and as described in previous literature, no patients experienced any significant side effects from topical losartan use and overall, there was quite a remarkable reduction in stromal haze. Future clinical trials should be conducted to assess the optimal onset, time course, and dosing of losartan treatment with and without other drops including topical steroids for different indications.

## Statement of Ethics

This study was performed in accordance with the Declaration of Helsinki. Ethical approval was not required for this study in accordance with local institutional guidelines for retrospective observational case reports. IRB Protocol number 2026P000793 was submitted to the Mass General Brigham IRB and approved on 6/1/2026 in “Active, Exempt” status. Written informed consent was obtained from all patients for publication of the details of their medical cases and any accompanying images. For patients under 18 years of age, written informed consent was obtained from their parent/legal guardian.

## Conflict of Interest Statement

The following authors have no financial disclosures: ECD, HS.

## Funding Sources

There was no funding or grant support provided for this research. It was performed as part of employment at Massachusetts Eye and Ear.

## Author Contributions

HS and ECD were responsible for reviewing medical records and writing the manuscript.

## Data Availability

All data generated or analyzed during this study are included in this article and its online supplementary material. Further inquiries can be directed to the corresponding author.
